# Vitamin E function and requirements in relation to PUFA

**DOI:** 10.1017/S000711451500272X

**Published:** 2015-08-21

**Authors:** Daniel Raederstorff, Adrian Wyss, Philip C. Calder, Peter Weber, Manfred Eggersdorfer

**Affiliations:** 1 DSM Nutritional Products, Basel CH-4002, Switzerland; 2 Human Development and Health Academic Unit, Faculty of Medicine, University of Southampton, Southampton SO16 6YD, UK

**Keywords:** Vitamin E, *α*-Tocopherol, PUFA, Requirements, Recommendations

## Abstract

Vitamin E (*α*-tocopherol) is recognised as a key essential lipophilic antioxidant in humans protecting lipoproteins, PUFA, cellular and intra-cellular membranes from damage. The aim of this review was to evaluate the relevant published data about vitamin E requirements in relation to dietary PUFA intake. Evidence in animals and humans indicates a minimal basal requirement of 4–5 mg/d of RRR-*α*-tocopherol when the diet is very low in PUFA. The vitamin E requirement will increase with an increase in PUFA consumption and with the degree of unsaturation of the PUFA in the diet. The vitamin E requirement related to dietary linoleic acid, which is globally the major dietary PUFA in humans, was calculated to be 0·4–0·6 mg of RRR-*α*-tocopherol/g of linoleic acid. Animal studies show that for fatty acids with a higher degree of unsaturation, the vitamin E requirement increases almost linearly with the degree of unsaturation of the PUFA in the relative ratios of 0·3, 2, 3, 4, 5 and 6 for mono-, di-, tri-, tetra-, penta- and hexaenoic fatty acids, respectively. Assuming a typical intake of dietary PUFA, a vitamin E requirement ranging from 12 to 20 mg of RRR-*α*-tocopherol/d can be calculated. A number of guidelines recommend to increase PUFA intake as they have well-established health benefits. It will be prudent to assure an adequate vitamin E intake to match the increased PUFA intake, especially as vitamin E intake is already below recommendations in many populations worldwide.

PUFA, categorised into *n*-3 and *n*-6 fatty acids, are important cell membrane components and key elements in child development, brain and visual functioning and physical health and well-being throughout the life course. Dietary sources that are rich in PUFA include many vegetable oils, nuts, seeds and certain types of fish. As PUFA contain double bonds, they are highly sensitive to oxidative stress; consequently, the oxidation of PUFA and the resulting lipid peroxides can have detrimental effects on development, brain function and human health. Normal metabolic, cell-signalling and host defence activities result in the release of oxidants and free radicals. If there are low concentrations of antioxidants present to counter-balance excessive concentrations of oxidants and free radicals – a situation often termed ‘oxidative stress’ – detrimental effects on cell components can occur^(^ ^1^ ^)^. The antioxidant defence system keeps the levels of oxidants and antioxidants balanced, and thus protects the body from the effects of oxidative stress^(^ ^1^ ^)^. In the human body, a complex network of antioxidant defence systems (mainly endogenous enzymatic antioxidant systems) is supported by, and interacts with, small antioxidant molecules derived from the diet to protect the tissues from oxidative stress. Vitamin E, or more specifically its *α*-tocopherol isoform, is one of the essential antioxidants that humans derive from the diet. Similar to PUFA, vegetable oils, nuts and seeds are particularly rich sources of vitamin E. Many nutrient databases and nutrition labels do not distinguish between the different isoforms of vitamin E, and often include the contribution of all eight naturally occurring vitamin E isoforms, presented as *α*-tocopherol equivalent. However, RRR-*α*-tocopherol is the isoform that is preferentially absorbed and maintained in the human body^(^ ^2^ ^,^ ^3^ ^)^. The Institute of Medicine (IoM), as well as other agencies that provide dietary intake recommendations – for example, the DACH-countries (Germany, Austria, Switzerland) – appreciate the role of vitamin E in protecting PUFA from being oxidised^(^ ^3^ ^,^ ^4^ ^)^. In the IoM 2000 report^(^ ^3^ ^)^, it is also noted that the amount of vitamin E needed to keep PUFA functional in cell membranes is obviously closely related to the intake of PUFA. As current vitamin E intakes are below the recommended intakes in more than 90 % of North Americans as well as in some European Countries^(^ ^5^ ^)^, and at the same time people are being encouraged to increase their intake of PUFA, especially those with high degree of unsaturation, because of their reported health benefits, the ratio of vitamin E:PUFA in the human diet appears to become more critical and requires a deeper examination. The aim of this study was to review the published evidence on the function and requirements of vitamin E:*α*-tocopherol in relation to the PUFA content of the human diet.

## Vitamin E

### Molecular structure and function of vitamin E

Vitamin E has eight isoforms; it can be categorised into tocopherol isoforms, which have a saturated side chain on the chromanol ring, and into tocotrienol isoforms, which have an unsaturated side chain. Each of these types is further categorised as *α*-, *β*-, *γ*- or *δ*-forms, which are defined by the number and the location of the methyl groups on the chromanol ring. The 6-hydroxy group of the chromanol ring is the active site for scavenging radicals, whereas the side chain does not affect the reactivity towards free radicals. Thus, all the isoforms of vitamin E have some antioxidant activity. However, other factors play a key role in determining whether a molecule has *in vivo* bioactivity; RRR-*α*-tocopherol has the highest *in vivo* bioactivity of all vitamin E isomers. It is bound by a specific transport protein – the *α*-tocopherol transfer protein (*α*-TTP). Thereby, it is protected within the cell and does not undergo rapid degradation processes like other vitamin E isoforms. Furthermore, *α*-TTP enables binding to the ABCA1 transporter and the secretion of RRR-*α*-tocopherol from hepatocytes to the periphery.

RRR-*α*-tocopherol is the only isoform of vitamin E that is essential for humans and it is considered the most important lipophilic antioxidant *in vivo*, in humans in particular, metabolising peroxyl radicals^(^ ^3^ ^)^. The eight vitamin E forms are all absorbed by humans but their degradation rate and retention time in the body differs widely, which directly impact their relative biopotency^(^ ^6^ ^,^ ^7^ ^)^. The bioactivity of the tocopherol and tocotrienol forms has been reviewed previously^(^ ^8^ ^,^ ^9^ ^)^. In short, the vitamin E forms are discriminated by the liver and only *α*-tocopherol is preferentially accumulated in the cellular membranes of tissues, whereas the other isoforms are rapidly metabolised and excreted in a similar manner as other xenobiotics^(^ ^6^ ^,^ ^9^ ^)^. *γ*-Tocopherol is the other vitamin E form that is present in significant amounts in the human diet as it is contained in a number of widely consumed vegetable oils^(^ ^9^ ^)^. *γ*-Tocopherol is slightly less efficient than *α*-tocopherol as a scavenger of oxygen radicals, but it is an efficient scavenger of reactive nitrogen species due to the unsubstituted 5-position on the chromanol ring^(^ ^9^ ^–^ ^11^ ^)^. However, *γ*-tocopherol is efficiently metabolised by cytochrome P450 enzymes. This may be the reason why even after intake of high doses of *γ*-tocopherol, its plasma concentration rarely exceeds 10 % of that of *α*-tocopherol, and much less is found in tissues^(^ ^9^ ^,^ ^12^ ^–^ ^18^ ^)^. *α*-Tocotrienol has been shown to have higher or similar antioxidant capacity in *in vitro* model systems than *α*-tocopherol, depending on the assays used^(^ ^19^ ^–^ ^22^ ^)^. However, tocotrienols are present only in very low amounts in the human diet as relevant amounts are only found in palm oil, rice bran, oats and barley^(^ ^9^ ^,^ ^23^ ^)^. Moreover, the tocotrienols are more rapidly metabolised than the corresponding tocopherol forms and levels of tocotrienols in cell membranes are very low^(^ ^13^ ^,^ ^14^ ^,^ ^17^ ^,^ ^18^ ^)^. In the brain, which is very rich in highly unsaturated fatty acids, *α*-tocopherol represents 99·8 % of the vitamin E content and no tocotrienols are detected^(^ ^18^ ^)^. Thus, the higher activity of *α*-tocopherol as an essential nutrient *in vivo* is related partially to its dietary intake and partially to its selective retention time in the body relative to the other vitamin E isoforms^(^ ^9^ ^)^. Therefore, we will not discuss in detail the activity of vitamin E isoforms other than *α*-tocopherol, and we will use the term vitamin E to refer to *α*-tocopherol in the remainder of this article.

Due to its structure and physical–chemical properties, vitamin E is one of the key antioxidants found in nature. After reaction with peroxyl radicals, an *α*-tocopheroxyl radical is formed.
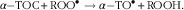
Due to the stability of the *α*-tocopheroxyl radical, it is unable to react further and the chain of oxidation events is broken. The chromanol ring is essential for the antioxidant power and the stability of the tocopheroxyl radical^(^ ^24^ ^,^ ^25^ ^)^.

Vitamin E is the key essential lipophilic (fat-soluble) antioxidant located in human cell membranes protecting them from oxidative damage. The essential role of vitamin E in the human antioxidant defence system has been re-evaluated by the European Food Safety Authority expert panel, which concluded that the scientific evidence indicates that ‘Vitamin E contributes to the protection of cell constituents from oxidative damage’^(^ ^26^ ^)^. *In vivo* and *in vitro* studies have shown that vitamin E functions as a chain-breaking antioxidant acting to protect unsaturated lipids from peroxidation by scavenging peroxyl radicals^(^ ^27^ ^)^. Animals and humans with a low vitamin E status are sensitive to muscle, neurological and embryogenesis problems that have been related to the protective effects of vitamin E against damage to PUFA in cell membranes and confirms its role as an essential nutrient^(^ ^7^ ^,^ ^28^ ^–^ ^30^ ^)^. A recent study in pregnant women observed that the rate of miscarriages was significantly increased in women having a low concentration of *α*-tocopherol (<12 µmol/l) in plasma^(^ ^31^ ^)^. These findings are in line with the numerous animal studies showing an essential role of *α*-tocopherol in embryonic development and clearly indicate the importance of an adequate intake of vitamin E in women of childbearing age. Clinical studies using pharmacological doses of vitamin E yielded conflicting results on the effects of vitamin E on CVD and optimal dosage and combination with other antioxidants still need to be determined^(^ ^32^ ^–^ ^35^ ^)^. However, epidemiological studies suggest a beneficial association between plasma vitamin E concentrations and CHD risk in individuals with an intake of vitamin E in the nutritional physiological range and a plasma *α*-tocopherol level >20 µmol/l with an optimum plasma level of about 30 µmol/l^(^ ^36^ ^–^ ^39^ ^)^.

### Interactions between vitamin E and PUFA

The incorporation of vitamin E into the membrane leads to stabilisation, and thus to a decrease in membrane fluidity. Only *α*-tocopherol, and not *β*-, *γ*- or δ-tocopherol, seems to have this effect. It was hypothesised for many years that *α*-tocopherol accumulates in lipid rafts where it stabilises the domain together with cholesterol. In recent years, a different theory was published^(^ ^40^ ^)^ and supported by studies in model membranes; vitamin E accumulates in DHA-rich, rather unstructured domains, where it exerts its main functions: stabilisation of the membrane and protection of DHA from oxidative damage. *α*-Tocopherol may, therefore, have a similar function as cholesterol in raft domains and may stabilise the PUFA-rich non-raft domains. In this way, the functional and structural roles of *α*-tocopherol would be combined in the same membrane area.

**Fig. 1.**
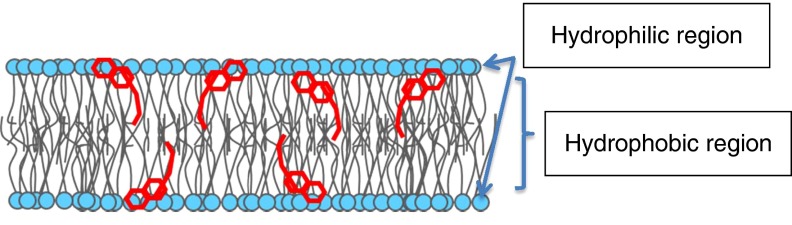
*α*-Tocopherol localisation in a membrane lipid bilayer.

Atkinson *et al*.^(^ ^40^ ^)^ undertook a series of biophysical experiments to describe the position of ^2^H-labelled *α*-tocopherol in the cell membrane. They concluded that the *α*-tocopherol molecule stands upright in the membrane bilayer (Fig. 1). The non-*α*-tocopherol isoforms can also be incorporated into cellular membranes. Studies in lipid model membranes indicate that the localisation of the various vitamin E forms in the membranes can slightly differ and they might alter the membrane behaviour differently due to their structural differences^(^ ^20^ ^,^ ^41^ ^)^. However, as mentioned previously, all non-*α*-tocopherol isoforms are rapidly metabolised and excreted and only *α*-tocopherol is present in significant amounts in cell membranes. Moreover, the biophysical studies assessing the localisation and the behaviour of vitamin E in membrane models have been carried out mainly with *α*-tocopherol^(^ ^41^ ^)^. The label at the chromanol ring is placed in the neighbourhood of the phospholipid glycerol backbone. The second label at the C9' of the side chain is located in the centre of the membrane, where the label can be found in various positions. This points to the *cis*-conformations rather than the extended all-*trans*-isomer. This localisation in the membrane suggests that the *α*-tocopherol antioxidant activity occurs at the membrane surface^(^ ^42^ ^)^. This location of vitamin E in the membrane would also allow vitamin C, which is placed at the hydrophilic/hydrophobic interphase, to interact with the *α*-tocopheroxyl radical and to bring it to the energetic ground state. This is the mechanism by which vitamin E is regenerated and is ready to interact with the next peroxyl radical^(^ ^7^ ^)^. Thus, both lipophilicity and membrane localisation of vitamin E explain its antioxidant activity.

The presence of vitamin E is of key importance in cellular membranes rich in highly unsaturated fatty acids such as DHA and arachidonic acid (AA), which are found in high concentrations in the brain, the retina and some other locations. Thus, in *α*-TTP-null mice fed a vitamin E-deficient diet, retinal structure is altered and lipid peroxidation is enhanced, whereas the concentration of DHA, the most abundant PUFA in the retina, is decreased^(^ ^43^ ^)^. In zebrafish, a model organism for lipid metabolism, vitamin E deficiency led to a decrease in the content of highly unsaturated fatty acids^(^ ^44^ ^)^, perhaps because of their consumption by peroxidative processes. The incorporation of *α*-tocopherol into membranes may be especially critical in early life, as DHA is required for optimal visual and brain development. Zebrafish embryos deficient in vitamin E develop morphologic abnormalities and have increased mortality, which is in line with a key role for vitamin E in fetal development^(^ ^45^ ^)^. In a recent study, Lebold *et al.* ^(^ ^46^ ^)^ showed that in zebrafish embryos *α*-tocopherol deficiency induced a decrease in DHA and AA concentrations during embryogenesis. In the adult zebrafish model, low brain *α*-tocopherol levels were associated with the depletion of DHA-containing phospholipids^(^ ^47^ ^)^. Those studies support the role of vitamin E in protecting PUFA in membranes in order to maintain optimal cellular function.

### New function of vitamin E in the membrane

Cellular membranes, especially those in muscle cells, are under constant physical stress. It has been elegantly demonstrated *in vitro* and in excised mouse muscle that *α*-tocopherol improves muscle membrane repair and rescues myocytes from necrosis^(^ ^48^ ^)^. A recent study in rats showed that the plasma membrane repair capacity is impaired in skeletal muscle fibres when the animals are deprived of vitamin E^(^ ^49^ ^)^. Thus, in myocyte plasma membranes, the presence of vitamin E promoted membrane repair. This is evidence that suggests a new function for *α*-tocopherol in membrane repair. The molecular basis of this effect is not clear yet, but it could be that the prevention of lipid peroxidation is involved. Membrane repair mechanisms involve many membrane fusion events. These processes might be much faster and more efficient in an antioxidant environment. Further, it was demonstrated in the same study that repair incompetent cells – that is, HeLa cells – could be rescued from laser-induced membrane damage by addition of *α*-tocopherol to the medium. These new findings may have an impact on future approaches against muscle pain – that is, concomitant after statin use – or support therapies for muscle injuries.

## PUFA

PUFA are involved in a wide range of processes that are related to physical and mental health. For example, *n*-3 PUFA play a role in cardiovascular health through multiple effects on the cardiovascular system, particularly on blood lipid concentrations, blood pressure and heart function^(^ ^50^ ^)^. Recent studies, systemic reviews and meta-analysis have yielded conflicting results with regard to the effect of *n*-3 PUFA on CVD. Some meta-analyses showed little or no effect of *n*-3 PUFA on cardiovascular events or cardiac death^(^ ^51^ ^–^ ^53^ ^)^, while others showed a benefit^(^ ^54^ ^–^ ^56^ ^)^. The reasons for these inconsistencies have been discussed in several studies^(^ ^57^ ^–^ ^61^ ^)^. It is to be noted that in most studies the background dietary intake of *n*-3 PUFA and the *n*-3 PUFA status at baseline were not taken into consideration. The outcome of meta-analyses investigating the same or a similar question can vary due to differences in methodology and the criteria used to select the relevant clinical studies for inclusion. Moreover, the selected studies are often very heterogeneous differing in sample size, design, population characteristics, type and dose of supplements used, end points measured and duration of the study, which may contribute to inconclusive and conflicting results. Thus, the results of such meta-analyses must be interpreted critically and have to be considered with caution. Overall, the risk benefit balance is still in favour of a benefit of *n*-3 PUFA^(^ ^60^ ^,^ ^61^ ^)^. Moreover, population studies still consistently show that a low *n*-3 PUFA (EPA and DHA) status is associated with an increased risk of CVD and cardiac death^(^ ^50^ ^,^ ^57^ ^,^ ^62^ ^)^. Finally, a number of cardiac societies, scientific and governmental organisations recommend the intake of *n*-3 PUFA for heart health^(^ ^63^ ^–^ ^67^ ^)^. Collectively, the data from epidemiological studies and clinical interventions still indicate that diets high in *n*-3 PUFA (EPA and DHA) reduce the risk of cardiovascular morbidity and mortality.

It has been suggested that adults with cardiac problems might benefit from *n*-3 PUFA, as they prevent atrial fibrillation recurrence and increase left ventricle systolic function and functional capacity^(^ ^68^ ^,^ ^69^ ^)^. A dose-dependent benefit of *n*-3 PUFA, whereby a higher dose was more beneficial, was demonstrated in a small trial on patients with chronic heart failure^(^ ^70^ ^)^. A systematic review showed that *n*-3 PUFA derived from oily fish were beneficial for patients with rheumatoid arthritis^(^ ^71^ ^)^. PUFA intake during pregnancy is important for fetal development and long-term development of children^(^ ^72^ ^)^. Furthermore, maternal *n*-3 PUFA intake during pregnancy may decrease the risk of allergies^(^ ^73^ ^)^ and improve bone health^(^ ^74^ ^)^ in early childhood. The involvement of *n*-3 and *n*-6 PUFA in mental health, particularly depressive moods, remains unclear but is of interest. A recent systematic review concluded that, although evidence is scarce, *n*-3 PUFA might be beneficial for those with diagnosed depression, whereas it has not been shown to be beneficial for those without diagnosed depressive illness^(^ ^75^ ^)^.

Finally, it is important to consider that the *n*-6:*n*-3 PUFA ratio is important, as a high ratio has been associated with higher risk of some diseases. For example, a high *n*-6:*n*-3 PUFA ratio has been associated with an increased risk of prostate cancer^(^ ^76^ ^)^. A review article by Patterson *et al.* ^(^ ^77^ ^)^ also implicated an association between high *n*-6:*n*-3 PUFA ratios and increases in chronic inflammatory disease, CVD, obesity, inflammatory bowel disease, rheumatoid arthritis and Alzheimer’s disease. All these reported health benefits of *n*-3 PUFA have resulted in guidelines and recommendations to increase daily *n*-3 PUFA intakes.

## Optimal balance between vitamin E and PUFA

### Animal studies

Harris & Embree^(^ ^78^ ^)^ reviewed a number of animal experiments, in which the animals were fed diets containing various vitamin E:PUFA ratios, which induced vitamin E deficiency or relieved it. Based on their analyses, which also included data on fat and *α*-tocopherol content in various food groups, they concluded that a vitamin E:PUFA ratio of 0·6 mg RRR-*α*-tocopherol for each gram of PUFA is necessary to protect against vitamin E deficiency. Following an experimental study in monkeys, Bieri & Poukka Evarts^(^ ^79^ ^)^ estimated that the minimum requirement was slightly >0·36 mg RRR-*α*-tocopherol/g of linoleic acid consumed and that 0·72 mg RRR-*α*-tocopherol/g of linoleic acid was nutritionally adequate.

The relationship between vitamin E requirements and the degree of unsaturation of PUFA was evaluated in young rats fed various fats with a constant total unsaturation but differing in the type of unsaturated fatty acid^(^ ^80^ ^)^. The time of onset of creatinuria was measured. The authors concluded that the relative quantities of *α*-tocopherol required to protect one mole of mono-, di-, tri-, tetra-, penta- and hexaenoic fatty acids were to be approximately in the ratios of 0·3:2:3:4:5:6, respectively. The findings are consistent with the *in vitro* susceptibility of unsaturated fatty acids to oxidative damage^(^ ^80^ ^)^.

### The Elgin project (1953–1967)

Vitamin E:PUFA ratios derived from animal studies might not be necessarily directly applicable for humans. The Elgin project, conducted between 1953 and 1967, was pivotal to evaluate the vitamin E requirement of humans through a long-term dietary study^(^ ^81^ ^–^ ^84^ ^)^. The adequacy of vitamin E intake was evaluated in the project by following plasma *α*-tocopherol levels over time. The susceptibility of erythrocytes to *in vitro* peroxide-induced haemolysis was considered a sign of vitamin E deficiency.

Three groups of men received different diets during the long-term follow-up. The first group received a basal diet consisting of 9204·8 kJ (2200 kcal), 47 g protein and 60 g fat, which was low in vitamin E (3–4 mg) and PUFA (9 g) each day. The second group received the same basal diet, low in vitamin E and PUFA, but supplemented with 15 mg/d of RRR-*α*-tocopherol. The third group was a control group that received a standard hospital diet *ad libitum*. The main fat component of the basal diet was 30 g/d of stripped lard, which was replaced by 30 g/d of stripped maize oil after 30 months to increase the dietary PUFA intake, which was increased to 60 g/d of maize oil another 9 months later to further increase the amount of PUFA.

The hospital diet providing 8–12 mg/d of *α*-tocopherol and 4–7 g/d of PUFA was considered as being on the borderline of adequacy over the 13 years of observation based on the plasma *α*-tocopherol levels and the data from the erythrocyte haemolysis test^(^ ^83^ ^)^.

The plasma *α*-tocopherol levels of the subjects in the basal diet group gradually decreased to around 12 µmol/l during the first 20 months. Moreover, the percentage of erythrocyte haemolysis was increased to levels above 20 % when the plasma *α*-tocopherol levels fell below 16 µmol/l after 7 months. These data show that a daily intake of 3–4 mg of *α*-tocopherol is inadequate^(^ ^81^ ^)^. Depleted subjects, who had been on the basal diet for 54 months, were given an *α*-tocopherol supplement of 15 mg/d for 138 d. However, this was insufficient to bring the plasma *α*-tocopherol levels and the erythrocyte haemolysis test back to baseline levels. Subjects receiving a supplement of 15 mg/d *α*-tocopherol in addition to the basal diet maintained a plasma *α*-tocopherol level around 23 µmol/l in the first 30 months of the study. The increase up to 60 g/d maize oil at 39 months lowered the plasma *α*-tocopherol levels, and a supplementation of 15 mg/d *α*-tocopherol proved insufficient to counteract this decrease. Therefore, the *α*-tocopherol supplement was increased to 30 mg/d^(^ ^82^ ^)^. Withdrawal of the *α*-tocopherol supplement led to an immediate drop in plasma *α*-tocopherol levels and a progressive increase in the erythrocyte haemolysis test. Finally, the *α*-tocopherol requirement in the absence of dietary PUFA was assessed by feeding subjects 60 g/d of beef tallow (a saturated fat) for 5 years. The diet provided about 2·4 g of PUFA and 3 mg of *α*-tocopherol daily. Plasma *α*-tocopherol levels decreased, whereas the sensitivity of erythrocytes to haemolysis increased. These data indicated that a minimal intake of 4–5 mg/d of *α*-tocopherol was needed in the basal state, even in the absence of dietary PUFA.

Collectively, the data from the Elgin project suggest that individuals ingesting large amounts of linoleic acid (>30 g/d) require more than 30 mg/d of *α*-tocopherol, whereas 10 mg/d of *α*-tocopherol may be bordering on inadequacy in individuals ingesting about 4–7 g/d of linoleic acid^(^ ^83^ ^)^. It was proposed that the *α*-tocopherol requirements in humans range from 10 to 30 mg/d depending on the amount of PUFA in the diet and the tissues^(^ ^83^ ^)^.

Horwitt^(^ ^83^ ^)^ proposed a calculation to quantify the vitamin E requirement in humans. This calculation was based on the sum of a basal minimum of 4 mg/d of *α*-tocopherol for normal cellular synthesis and retention of PUFA in membranes to which a factor was added that depended on the percentage of dietary PUFA and the grams of PUFA consumed:
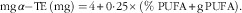
In another analysis, the vitamin E requirement in relation to PUFA was estimated as the sum of a basal requirement of 5 mg/d *α*-tocopherol to which 0·5 mg *α*-tocopherol for each gram of PUFA is to be added^(^ ^84^ ^)^:
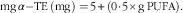
Based on the evaluation of the human data from the Elgin project, the minimum vitamin E:PUFA ratio to avoid the development of vitamin E deficiency symptoms is between 0·5 and 0·8 mg *α*-tocopherol/g of PUFA^(^ ^78^ ^,^ ^85^ ^)^. However, the validity of the erythrocyte haemolysis test to define the vitamin E requirement in relation to PUFA intake, which was used as an early sign of vitamin E deficiency, has been questioned^(^ ^85^ ^)^. Therefore, basing vitamin E:PUFA ratio only on data from the Elgin project may not be appropriate.

### Other human studies

Using data from the Second National Health and Nutrition Examination Survey, Murphy *et al.* concluded that the dietary vitamin E:PUFA ratio decreased with increased PUFA intake from 0·94 for individuals with diets low in PUFA (<5 g/d) to 0·44 for individuals with PUFA intakes >25 g/d. Earlier studies reported a ratio of 0·43 based on the analysis of composite meals^(^ ^86^ ^)^ and of 0·52 based on an experimental diet providing an adequate intake of vitamin E^(^ ^87^ ^)^. A ratio of 0·4 has been found sufficient to maintain plasma vitamin E levels in growing children in another study^(^ ^88^ ^)^. Similarly, Witting & Lee^(^ ^89^ ^)^ reported that a ratio of approximately 0·4 mg RRR-*α*-tocopherol/g of linoleic acid was sufficient in young women, following a 9-month study period. The above-mentioned human studies have mainly focused on the relationship between vitamin E and intake of linoleic acid, the major PUFA in western diets. However, only a few studies have evaluated the relation and interactions between vitamin E intake and the consumption of highly unsaturated fatty acids such as EPA and DHA in humans. In several studies, the effect of an increased intake of *n*-3 PUFA from fish on vitamin E status and lipid peroxidation was investigated with inconsistent results. In a few studies, the increase in lipid peroxidation induced by a high intake of *n*-3 PUFA could not be prevented by an increased intake of vitamin E^(^ ^90^ ^,^ ^91^ ^)^. However, in other studies, the rise in lipid peroxidation after the intake of *n*-3 PUFA could be overcome by a higher intake of vitamin E^(^ ^92^ ^–^ ^94^ ^)^. The different findings may be due to difference in the methodologies used to measure the oxidative stress markers or interactions with other dietary factors. Moreover, those studies that used pharmacological doses of both *n*-3 PUFA and vitamin E are difficult to extrapolate to the general population. Recently, an epidemiological study investigating the association between PUFA intake and C-reactive protein (CRP) concentration found potential interactions with vitamin E intake^(^ ^95^ ^)^. However, these interactions were only significant for EPA and DPA, which are present in low amounts in tissues, whereas no significant interactions were found for the major *n*-6 and *n*-3 PUFA found in cell membranes such as linoleic acid, AA and DHA. Thus, it seems unlikely that this interaction is related to the PUFA-protective role of vitamin E in cellular membranes. Moreover, the associations found in this study need to be confirmed by additional studies. In some studies, vitamin E treatment has been suggested to decrease CRP levels in humans^(^ ^96^ ^)^. Therefore, it may be that with an adequate intake of vitamin E no additional improvement may actually be observed by an increase in the PUFA intake. Interestingly, a low and moderate intake of DHA (200, 400 mg/d) in humans reduced oxidative stress and increased plasma and platelet vitamin E levels, whereas a high intake of DHA increased oxidative stress^(^ ^97^ ^,^ ^98^ ^)^. The conflicting results obtained in previous studies clearly indicate that additional studies are needed, probably using novel methodology to measure the interactions between vitamin E and *n*-3 PUFA, in order to further clarify the relationship and interactions between vitamin E intake and the consumption of highly unsaturated fatty acids.

### Estimating vitamin E:PUFA requirements

The estimated optimal vitamin E:PUFA ratio seems to be relatively consistent across studies. Taking these studies together suggests an estimated additional vitamin E requirement ranging from 0·4 to 0·6 mg RRR-*α*-tocopherol/g of PUFA in the diet for a diet with a typical content of PUFA, mainly as linoleic acid. A ratio of 0·5 mg RRR-*α*-tocopherol/g of PUFA in the middle of this range may reasonably be used to calculate the vitamin E requirement. Thus, considering a basal requirement of at least 4 mg RRR-*α*-tocopherol as suggested by the human data from the Elgin project, the following formula can be used to calculate the vitamin E requirement: vitamin E requirement=4+(0·5× amount of PUFA in the diet in grams). However, the vitamin E requirement also depends on the degree of unsaturation of PUFA in the diet^(^ ^80^ ^)^, and most of the studies described so far have considered linoleic acid as the main dietary PUFA. Therefore, Muggli^(^ ^99^ ^)^ proposed to estimate the dietary vitamin E requirement by taking the relative vitamin E requirement for individual PUFA into account. The vitamin E demand for individual PUFA found in the human diet can then be calculated based on the vitamin E:linoleic acid ratio (milli grams *α*-tocopherol per gram of linoleic acid) extrapolated from human studies and the relative vitamin E requirement for individual PUFA (0·3:2:3:4:5:6 for, respectively, mono-, di-, tri-, tetra-, penta- and hexaenoic fatty acids) extrapolated from animal data. Table 1 shows the vitamin E requirement for individual PUFA using a vitamin E:linoleic acid ratio of 0·5 mg *α*-TE/g of linoleic acid in the diet.

**Table 1:** Vitamin E requirements for different unsaturated fatty acids found in human diets

Number of double bonds	Fatty acid	Vitamin E requirement (mg/g fatty acid)
1	Oleic acid	0·075
2	Linoleic acid	0·5
3	*α*-Linolenic acid	0·75
4	Arachidonic acid	1·0
5	EPA	1·25
6	DHA	1·5

It should be noted that the studies that estimated the additional requirement of vitamin E in relation to dietary PUFA were conducted with diets that were very rich in PUFA or containing linoleic acid almost exclusively. Thus, the reported ratio of vitamin E:PUFA of 0·4–0·6 mg RRR-*α*-tocopherol/g of PUFA from these studies may not be relevant for ratios with other PUFA with more than two double bonds. Consequently, considering a basal requirement of 4 mg of RRR-*α*-tocopherol, the following formula could be used to calculate the vitamin E requirement, in which Mn is the amount of dietary PUFA with *n* double bonds in grams:
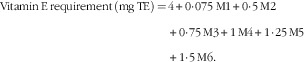
M1 to M6 are expressed in gram of the respective unsaturated fatty acids.

Furthermore, in order to quantify the vitamin E requirement, one needs to estimate the typical intake range for PUFA with different degree of unsaturation (Table 2). The PUFA intake range indicated in Table 2 was estimated from reviews providing a global perspective on dietary fatty acid intake^(^ ^100^ ^–^ ^102^ ^)^. The recent worldwide analysis of dietary fats and oils consumption from Micha *et al.* ^(^ ^101^ ^)^ indicates that there are large variations across regions and countries in the consumption of fats and oils, and therefore of fatty acids including PUFA. The mean global *n*-6 PUFA (mainly linoleic acid) intake was 5·9 % energy ranging from 2·5 to 8·5 % energy across regions. The typical linoleic acid intake in a western population would range from about 12 to about 21 g/d, considering an energy intake ranging from 7531·2 to 13 455 kJ/d (1800 to 3250 kcal/d). The worldwide mean plant *n*-3 PUFA (mainly *α*-linolenic acid) intake was 1371 mg/d with a 10-fold variation from 302 to 3205 mg/d across regions. The worldwide ‘seafood’ *n*-3 PUFA (mainly EPA and DHA) intake was 163 mg/d, ranging widely from <50 to >700 mg/d between regions.

**Table 2:** Estimated vitamin E requirement for typical ranges of unsaturated fatty acid intake in western diets

Unsaturated fatty acid	Typical intake range (g/d)	Vitamin E requirement (mg/g fatty acid)
Oleic acid	20–30	1·5–2·3
Linoleic acid	12–21	6·0–10·5
*α*-Linolenic acid	1·1–2·6	0·8–2·0
Arachidonic acid	0·1–0·3	0·1–0·3
EPA+DHA	0·1–0·5	0·1–0·7
Vitamin E requirement related to unsaturated fatty acid intake		8·5–15·7 mg/d
Total vitamin E requirement		12·5–20 mg/d*

* The estimation is based on the vitamin E requirement related to unsaturated fatty acid intake plus a basal requirement of 4 mg of RRR-*α*-tocopherol/d.

## Vitamin E recommendations

It is recommended that a baseline *α*-tocopherol requirement be estimated to which an additional vitamin E intake to compensate for dietary PUFA intake can be added in order to obtain the correct balance of dietary fatty acids with vitamin E. This baseline vitamin E requirement would allow for adequate synthesis and selective storage of PUFA in cell and tissue lipids, even with very low-PUFA diets. However, the optimal vitamin E requirement also depends on the amount and degree of unsaturation of PUFA in the diet. Increased dietary intake of PUFA has been shown to decrease vitamin E levels in plasma and tissues in both animals and humans. Moreover, high intake of PUFA with concomitant very low intake of vitamin E may lead to symptoms of vitamin E deficiency such as creatinuria, erythrocyte fragility and increased lipid peroxidation. However, it has been difficult to determine the precise vitamin E requirements in humans, as there are apparently no clear deficiency diseases. Furthermore, very long-term follow-up is needed to deplete the body stores of vitamin E to investigate any long-term deleterious consequences, which are often difficult to diagnose at an early stage. However, at present, it would not be feasible to conduct such long-term follow-up or depletion studies, such as the Elgin project, due to ethical considerations.

### Institute of Medicine and DACH recommendations

In 2000, the IoM^(^ ^3^ ^)^ published dietary reference intakes for vitamin E, based on previously reported research findings. The report suggested that there was no scientific basis to assume any difference in vitamin E requirements between men and women, or that ageing impairs vitamin E absorption or secretion. Therefore, no distinction was made between sexes or adult age categories in the IoM recommendations.

In the same year, the DACH reference values^(^ ^4^ ^)^ were published for Germany, Austria and Switzerland, using similar methodology as the IoM, but based on relevant German, Austrian and Swiss data. In contrast to the IoM reference values, the DACH values distinguish between sexes and adult age categories. Reference values from the IoM and DACH reports are shown in Table 3.

**Table 3:** Estimated daily vitamin E intake (mg) as reported in the Institute of Medicine (IoM) and DACH recommendations

Age range	Estimated daily intake based on EAR according to IoM	Estimated daily intake according to DACH*
0–3 months	–	3
0–6 months	4†	–
4–12 months	–	4
7–12 months	5†	–
1–3 years	5	Male: 6, female: 5
4–6 years	–	8
4–8 years	6	–
7–9 years	–	Male: 10, female: 9
9–13 years	9	–
10–12 years	–	Male: 13, female: 11
13–14 years	–	Male: 14, female: 12
14–18 years	12	–
15–18 years	–	Male: 15, female: 12
>19 years	12	–
19–24 years	–	Male: 15, female: 12
25–50 years	–	Male: 14, female: 12
51–64 years	–	Male: 13, female: 12
>65 years	–	Male: 12, female: 11
Pregnant women	12	13
Lactating women	16	17

EAR, estimated average requirement; DACH, Germany, Austria, Switzerland.

* Estimated intake based on adequate intake in a diet principally consisting of human milk.

† Equivalent to EAR.

Both reference value sets distinguish between pregnant and lactating women. The IoM found no evidence to believe that pregnant women require an increased vitamin E intake, compared with non-pregnant women of the same age, whereas the DACH values are slightly increased for pregnant women. Both reference sets suggest an increased vitamin E intake for lactating women, to account for the estimated 4 mg/d of vitamin E secreted via human milk.

Although there are only slight differences between the IoM and DACH reference values, they used two different methodological approaches to propose these recommendations for dietary vitamin E intake. The IoM based their recommendation mainly on the prevention of deficiency symptoms, using the sensitivity of erythrocytes to haemolysis as a sign of deficiency. The available human data indicate that individuals with a plasma concentration of at least 12 µmol/l of *α*-tocopherol have a low per cent of erythrocyte haemolysis. It is of interest to note that this plasma level of vitamin E is in line with the recent findings, indicating an increased risk of miscarriage in women with a plasma vitamin E concentrations <12 µmol/l^(^ ^31^ ^)^. A plasma *α*-tocopherol level of 12 µmol/l was estimated to correspond to an intake of about 12 mg/d. Thus, the estimated average requirement (EAR) for vitamin E was set to 12 mg/d of *α*-tocopherol to prevent erythrocyte haemolysis. A RDA of 15 mg/d of *α*-tocopherol for both men and women was calculated from the EAR plus twice the CV (10 %) to take into account the individual needs.

The DACH recommendations considered the dietary PUFA intake to estimate the vitamin E requirement by taking into account a basal vitamin E requirement plus an additional requirement based on the dietary intake of PUFA. To calculate the vitamin E requirement, they used a basal vitamin E requirement of 4 mg/d and a ratio of 0·4 mg of *α*-tocopherol/g of dietary linoleic acid and the ratios between vitamin E and dietary PUFA with different degree of unsaturation proposed by Witting & Horwitt^(^ ^80^ ^,^ ^83^ ^,^ ^89^ ^)^. The dietary vitamin E requirements for adult women and men were estimated to be respectively 12 and 15 mg/d based on a typical dietary intake of PUFA, which differ between women and men due to difference in energy intake. This is in line with the approach we have used in this review to estimate the requirement for vitamin E in relation to dietary PUFA. It is interesting to note that, despite using two different methods, both approaches lead to reference values that fall within the 10–30 mg/d range as suggested in a previous study.

Several publications and reviews have discussed the quantification of the physiological requirement of vitamin E as a function of PUFA intake^(^ ^85^ ^,^ ^86^ ^,^ ^99^ ^,^ ^103^ ^,^ ^104^ ^)^. The authors suggested various ratios of vitamin E to PUFA expressed as milligram tocopherol per gram to calculate the additional vitamin E requirement as a function of the amount of PUFA in the diet. Moreover, the data indicate that based on animal and human studies an attempt to quantify the vitamin E requirement should take two factors into account: (1) the minimum requirement to allow for basal metabolism, cellular synthesis and PUFA retention, even in low-PUFA diets, and (2) the additional vitamin E required to protect and metabolise dietary PUFA.

### Considerations for future recommendations

From Table 3 and previously published studies, it becomes evident that recommendations for vitamin E and PUFA intake are developed and reported in various ways. The main differences are the categories and methods that are used to estimate vitamin E requirements. IoM did not distinguish between sexes, whereas DACH did. Furthermore, both reports used different age categories, which was likely the result of the age categories provided in the referenced studies. In order to allow for better comparison across studies and reports, we would like to suggest that future studies report their recommendations using standardised age categories when possible. In addition, we suggest the inclusion of an aggregate reference value for males and females combined. Finally, we recommend a standardised method for estimating vitamin E requirements.

## Conclusions

The main function of vitamin E is to protect lipids from oxidative damage, and a number of studies show that its requirement is related to the dietary intake of PUFA. Thus, to quantify the vitamin E requirement, a basal vitamin E requirement plus an additional vitamin E requirement for dietary PUFA is often considered. A precise vitamin E:PUFA ratio may not be applicable to all types of diet and health status. Therefore, there has been no consensus on the exact vitamin E:PUFA ratio to determine the vitamin requirement. However, the published human data for a diet with an average content of PUFA and containing mainly linoleic acid as the PUFA indicate that the additional vitamin E requirement ranges from 0·4 to 0·6 mg RRR-*α*-tocopherol/g of PUFA in the diet. We used a ratio of 0·5 mg RRR-*α*-tocopherol/g of linoleic acid in the diet and also considered the degree of unsaturation of the dietary fatty acids to evaluate the vitamin E required. Thus, using the proposed equation, the estimated requirement for vitamin E varied from 12 to 20 mg/d for a typical range of dietary PUFA intake. Although more research is needed to precisely define the vitamin E requirement in humans, it is important in view of the critical interactions for health between vitamin E and PUFA to ensure an adequate intake of vitamin E in humans, particularly when the dietary PUFA intake is increased.
